# Epithelial Cell Inflammasomes in Intestinal Immunity and Inflammation

**DOI:** 10.3389/fimmu.2017.01168

**Published:** 2017-09-20

**Authors:** Andrea C. Lei-Leston, Alison G. Murphy, Kevin J. Maloy

**Affiliations:** ^1^Sir William Dunn School of Pathology, University of Oxford, Oxford, United Kingdom

**Keywords:** inflammasome, IL-18, IL-1β, intestinal epithelial cells, NOD-like receptor, pyroptosis, inflammatory bowel disease

## Abstract

Pattern recognition receptors (PRR), such as NOD-like receptors (NLRs), sense conserved microbial signatures, and host danger signals leading to the coordination of appropriate immune responses. Upon activation, a subset of NLR initiate the assembly of a multimeric protein complex known as the inflammasome, which processes pro-inflammatory cytokines and mediates a specialized form of cell death known as pyroptosis. The identification of inflammasome-associated genes as inflammatory bowel disease susceptibility genes implicates a role for the inflammasome in intestinal inflammation. Despite the fact that the functional importance of inflammasomes within immune cells has been well established, the contribution of inflammasome expression in non-hematopoietic cells remains comparatively understudied. Given that intestinal epithelial cells (IEC) act as a barrier between the host and the intestinal microbiota, inflammasome expression by these cells is likely important for intestinal immune homeostasis. Accumulating evidence suggests that the inflammasome plays a key role in shaping epithelial responses at the host–lumen interface with many inflammasome components highly expressed by IEC. Recent studies have exposed functional roles of IEC inflammasomes in mucosal immune defense, inflammation, and tumorigenesis. In this review, we present the main features of the predominant inflammasomes and their effector mechanisms contributing to intestinal homeostasis and inflammation. We also discuss existing controversies in the field and open questions related to their implications in disease. A comprehensive understanding of the molecular basis of intestinal inflammasome signaling could hold therapeutic potential for clinical translation.

## Introduction

Intestinal homeostasis is governed by complex interactions between the host immune system, the vast constitutive antigenic load in the lumen, and the epithelial barrier. Breakdown in this molecular dialog can lead to the development of chronic pathologies, such as inflammatory bowel diseases (IBD). The precise etiology of IBD remains unclear, although it is likely multifactorial involving a number of elements, such as host genetic susceptibility, environmental factors (e.g., smoking), and the composition of the microbiome (1). These factors contribute to the disturbance of homeostasis leading to the generation of chronic inflammation and development of IBD, including Crohn’s disease (CD) and ulcerative colitis (UC). IBD are debilitating, relapsing diseases affecting approximately 1:400 people. With no cure available, IBD patients are consigned to long-term anti-inflammatory and immune suppressive therapies, and surgery is often required. Thus, there is an urgent, unmet need to further understand the molecular mechanisms underlying IBD, to inform the development of new potential therapies. Genome-wide association studies (GWAS) revealed that inflammasome-associated genes were linked to IBD susceptibility (2), suggesting that this family of proteins is important for maintenance of intestinal homeostasis.

The inflammasome is a multimeric protein complex involved in inflammation. It comprised of an intracellular Pattern Recognition Receptors (PRR), usually a NOD-like receptor (NLR), and is activated in response to exogenous pattern-associated molecular patterns (PAMP) or endogenous danger-associated molecular patterns (DAMP) (3). NLR are highly conserved throughout evolution attesting to their important role in host defense (4). NLR possess three domains: the N-terminal effector domain that may be a caspase recruitment domain (CARD), a pyrin (PYD) domain, or a baculovirus inhibitor of apoptosis repeat (BIR) domain; the central nucleotide-binding oligomerization domain (NOD); and the C-terminal domain comprised of leucine rich repeat sequences (LRR) (5). Based on their N-terminal domains, NLR can be divided into four main families (Table 1). Different NLR have been linked to the detection of different signals, for example, NLRC4 recognizes bacterial flagellin (6, 7) whereas NLRP1 has been implicated in the sensing of anthrax lethal toxin (8), but the specific molecular ligands for a majority of NLRs remain uncharacterized. In some cases, the LRR of the C-terminal bind directly to the PAMP (5); however, the precise mechanism of agonist activation of NLR remains to be determined, as other reports have postulated an auto-inhibitory role for the LRR (9).

**Table 1:** NLR family members and other inflammasome components.

NLR/inflammasome component		Ligand/agonist	Expression in IEC
**NLR family**			

NLRA (acidic activation domain)	CIITA	Unknown	Yes (10, 11)

NLRB1 (BIR domain)	NAIP1, NAIP2	T3SS (12, 13)	Yes (14–16)
	NAIP5, NAIP6	Flagellin (12, 13)	Yes (14–16)

NLRC (CARD domain)	NLRC1 (NOD1)	iE-DAP (17)	Yes (18)
	NLCR2 (NOD2)	MDP (19, 20)	Yes (18, 21, 22)
	NLRC4	Flagellin, T3SS rod proteins (*via* NAIP) (6, 7, 23)	Yes (24–26)
	NLRC3 + 5	Unknown	ND

NLRP (PYRIN domain)	NLRP1	Anthrax lethal toxin, ATP, and MDP (8, 27)	Yes (28)
	NLRP3	ATP, MSU, toxins, oxidized mitochondrial DNA, alum, silica, UV radiation, amyloid β (5, 29), and SCFA (acetate) (30)	Yes (26, 31)
	NLRP6	Metabolites (e.g., taurine, spermine, and histamine) (32)	Yes (33–35)
	NLRP7	Microbial lipopeptides (36)	ND
	NLRP9b	dsRNA (37)	Yes (37)
	NLRP12	*Yersinia pestis* (38)	ND
	NLRP 2, 4, 5, 8, 10, 11, 13 + 14	Unknown	ND

Unclassified	NLRX1	ssRNA, dsRNA, and poly (I:C) (39)	Yes (40)

**Inflammasome components**			

AIM2		dsDNA (41)	Yes (42)
Asc		NA	Yes (16, 43)
Caspase-1		NA	Yes (26, 44, 45)
Human caspase-4/murine caspase-11		LPS (46)	Yes (44, 46–48)
Caspase-8		ND	Yes (24)
IL-1β		NA	Yes (44)
IL-18		NA	Yes (44, 49–52)

*SCFA, small chain fatty acids; ND, not determined; NA, not applicable; T3SS, type 3 secretion system; IEC, intestinal epithelial cells; CARD, caspase recruitment domain; AIM2, absent in melanoma 2; dsDNA, double-stranded DNA; NLR, NOD-like receptor*.

Upon sensing of endogenous or exogenous danger signals, some NLR oligomerize *via* their NOD domains. If the NLR contains a CARD domain this can facilitate the recruitment of the inactive enzyme pro-caspase-1, through direct CARD–CARD interactions. However, inflammasome-forming NLR lacking a CARD domain use their PYD domain to recruit the adaptor protein Apoptosis-associated speck-like protein containing CARD (Asc)—comprising a PYD and a CARD domain, and this serves as a scaffold, bridging the interactions between the NLR and pro-caspase-1. This “canonical” inflammasome formation results in the autocatalytic activation of caspase-1. Caspase-1 has two main functions, cleavage of pro-IL-1β and pro-IL-18 into their active forms for secretion (53, 54), and the induction of a specialized form of inflammatory cell death known as pyroptosis (55–57). Another form of inflammasome has been described which does not require a member of the NLR family, but instead contains members of the PYHIN family (PYD and HIN domain containing). For example, the PYHIN family member absent in melanoma 2 (AIM2) can directly bind to its stimulus, double-stranded DNA (dsDNA), which may be present in the cytosol during infection, to form a caspase-1 containing inflammasome (41).

**Figure 1:**
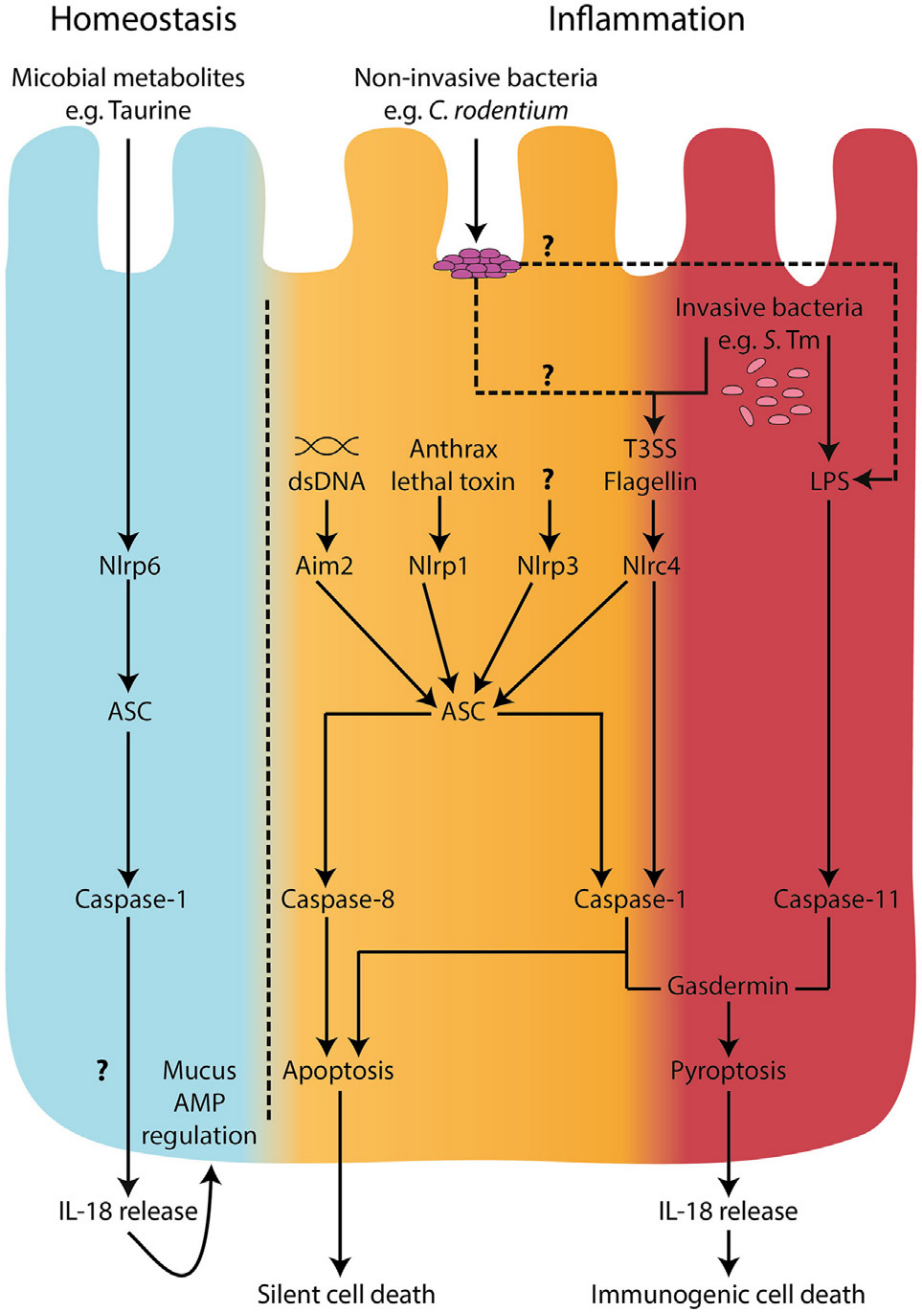
Inflammasomes in intestinal epithelial cells. During homeostatic conditions, in the absence of inflammation, IL-18 is released from epithelial cells and is involved in epithelial repair, proliferation, and maturation (33, 34). A metabolomics screen identified microbiome-derived metabolites, including taurine, that are capable of modulating NLRP6 inflammasome activation and subsequent IL-18 secretion (32). However, the mechanisms of release of IL-18 during homeostatic conditions are undefined. In the context of microbial invasion and pathogen-associated molecular pattern stimulation, inflammasome activation in intestinal epithelial cells has been described to engage both “canonical,” caspase-1-mediated and “non-canonical,” caspase-11 pathways (14, 24, 44). Recently, caspase-8 was also shown to be involved in inflammasome responses downstream of NLRC4 engagement with intracellular flagellin (24). Both caspase-1 and caspase-11 can lead to cell death by pyroptosis accompanied by IL-18 secretion; however, caspase-1 and caspase-8 were shown to lead to a non-lytic form of cell death upon NLRC4 sensing of intracellular flagellin (24). These observations raise the possibility of a distinction between a pro-immunogenic cell death signal driven by caspase-11 and GsdmD, a pro-silent cell death driven by caspase-8, and perhaps a threshold-dependent cellular decision between non-lytic and lytic forms of cell death involving caspase-1. Under low stress levels, it would be desirable to deal with the invading threat in an immunologically silent way. However, when the threat is high, an immunogenic cell death could recruit inflammatory cells to help clear the microbial insult.

Of emerging interest in the field is the formation of “non-canonical” inflammasomes by caspase-11 and caspase-8. Caspase-11 was originally discovered to be important in caspase-1 and -3 activation (58) and has been found to indirectly increase processing of pro-IL-1β and pro-IL-18 by promoting NLRP3 inflammasome activation (59). Indeed, it was shown that caspase-11 can detect intracellular LPS, and some intracellular bacteria, leading to cell death (60, 61). The human orthologs of murine caspase-11, namely, caspase-4 and -5, appear to serve similar functions (46, 62). Recently, an inflammasome formed by NLRC4, Asc, and potentially caspase-8 was described in a model of enteric *Salmonella enterica* serovar Typhimurium (*S*. Tm) infection, and this inflammasome was required for expulsion of infected intestinal epithelial cells (IEC) (Table 2) (24). There has also been a report of caspase-8 driving caspase-1 cleavage and downstream pro-IL-1β cleavage during *Yersinia pestis* infection of macrophages (63). Although immune cells and IEC express both “canonical” and “non-canonical” inflammasome components, how these complexes interact with one another upon stimulation and tailor their responses (e.g. pro-inflammatory cytokine secretion versus pyroptosis) remains to be elucidated (Figure 1).

**Table 2:** Inflammasome components and intestinal inflammation.

Mutant strain	Trigger	Effect	Reference
**Inflammasome components**			

Asc^−/−^	DSS	Increased pathology	(33, 42, 52, 131, 164)
		Decreased IL-18 levels	(33, 52)
		Decreased AMP levels Treatment with taurine rIL-18 ameliorated disease	(32)

	*C. rod*	Increased bacterial colonization	(34, 43, 103)
		Increased pathology	(43, 103)
		Decreased IL-18 levels	(43)
		Decreased mucus secretion by goblet cells	(34)

	Rotavirus	Increased viral load	(37)

Casp1^−/−^Casp11^−/−^	DSS	Increased pathology	(33, 51, 52, 164)
		Decreased IL-18 levels Phenotype rescued by rIL-18	(51, 52)

	*C. rod*	Increased bacterial colonization	(34)

	FlaTox	Decreased IEC pyroptosis	(24)

	NSAID-induced SI damage	Decreased pathology Decreased IL-1β levels	(165)

Caspase1^−/−^	DSS	Decreased pathology Decreased IL-18 levels	(142)

	Rotavirus	Increased viral load	(37)

Casp1^ΔIEC^	DSS	Decreased pathology Decreased IL-18 levels	(142)

Casp1^ΔIEC^	Rotavirus	Increased viral load	(37)

Caspase11^−/−^	DSS	Increased pathology	(47, 48)
		Increased IL-18	(48)
		Decreased IL-18 and IL-22 Phenotype rescued by rIL-18	(47)

	*S*. Tm	Decreased IL-18 levels Decreased pathology Increased intraepithelial bacterial burden Decreased IEC extrusion	(44)

gasdermin D^−/−^	FlaTox	Decreased IEC pyroptosis	(24)

gasdermin D^−/−^	Rotavirus	Increased viral load Decreased IEC death	(37)

Casp1^−/−^Casp8^−/−^Ripk3^−/−^	*S*. Tm FlaTox	Decreased IEC extrusion	(24)

**NLR proteins**			

NAIP1–6^Δ/Δ^	*S*. Tm	Increased intraepithelial bacterial loads Decreased IEC expulsion	(14)

NAIP1–6^Δ/ΔIEC^	*S*. Tm	Increased intraepithelial bacterial loads	(14)

NLRC4^−/−^	DSS	Increased pathology	(30)

	*C. rod*	Increased bacterial colonization Increased pathology Decreased IL-18 at steady state	(25)

	*S*. Tm	Increased intraepithelial bacterial loads	(14)

iNLRC4^+^Vil-Cre^+^	*S*. Tm FlaTox	Comparable bacterial burden Comparable IL-18 and PGE_2_ levels Comparable caspase-1 and caspase-8 activation	(24)

NLRP1^−/−^	DSS	Increased pathology Rescued by treatment with rIL-1β or rIL-18 or antibiotics	(131)

NLRP3^−/−^	DSS	Increased pathology	(30, 42, 52, 164)
		Decreased pathology Decreased IL-1β	(166)

	*C. rod*	Increased pathology	(43, 103)
		Increased bacterial colonization	(43, 103)

	T cell transfer colitis	Increased pathology upon transfer of NLRP3^−/−^ T cells into lymphopenic hosts Increased Th17 cells and decreased Th1 cells	(167)

	NSAID-induced SI damage	Decreased pathology Decreased IL-1β levels	(165)

NLRP6^−/−^	DSS	Increased pathology	(33)
		Decreased IL-18 levels	(32, 33)
		Decreased AMP levels	(32)

	*C. rod*	Increased bacterial colonization Decreased mucus secretion by goblet cells Decreased autophagosome formation	(34)

NLRP9b^−/−^	Rotavirus	Increased viral load Decreased IEC death	(37)

NLRP9b^ΔIEC^	Rotavirus	Increased viral load	(37)

NLRP12^−/−^	DSS	Increased pathology	(168–170)

NLRX1^ΔIEC^	DSS	No change in pathology Increased IEC proliferation	(40)

**PYHIN sensors**			

AIM2^−/−^	DSS	Increased pathology	(42, 129)
		Decreased IL-1β levels	(129)
		Decreased IL-18 levels	(42, 129)
		Decreased IL-22BP levels	(42)
		Dysregulated AMP levels	(42, 129)

*AMP, antimicrobial peptides; C. rod, Citrobacter rodentium; FlaTox, Legionella pneumophila flagellin fused to the N-terminal domain of Bacillus anthracis lethal factor; NAIP5, ligand delivered to cytosol; IEC, intestinal epithelial cells; NSAID, non-steroidal anti-inflammatory drugs; SI, small intestine; S. Tm, Salmonella Typhimurium; DSS, dextran sodium sulfate; rIL-18, recombinant IL-18; NLR, NOD-like receptor*.

*Mutant strain: Casp1^ΔIEC^, caspase-1-deficient IEC; NAIP1–6^Δ/ΔIEC^, NAIP1–6-deficient IEC; iNLRC4^+^Vil-Cre^+^, NLRC4 only expressed in IEC; NLRP9^ΔIEC^, NLRP9b-deficient IEC; NLRX1^ΔIEC^, NLRX1-deficient IEC*.

Innate immune recognition at mucosal surfaces, in particular the intestine, is a critical mediator of homeostasis (64). Indeed, in the gut, PRR sensing has been implicated in several key processes, such as maintenance and repair of the epithelial barrier and production of antimicrobial peptides (AMP) (65–67). Aside from basal roles at steady state, effective PRR signaling also protects against enteric pathogens by initiating immune responses Tables 2 and 3 (68–70). To date, the majority of work has focused on the role of the hematopoietic compartment in microbial detection and inflammation, but non-hematopoietic cells, particularly IEC, are now appreciated to be important contributors to PRR sensing circuits in the gut (71).

**Table 3:** Soluble mediators of inflammasome activation and intestinal inflammation.

Mutant strain	Trigger	Effect	Reference
**IL-1R1 signaling pathway**			

IL-1αβ^−/−^	*S*. Tm	No effect on intraepithelial bacterial load	(14)

IL-1β^−/−^	DSS	Decreased pathology Hematopoietic expression (monocytes)	(98)

	*C. rod*	Increased bacterial colonization Increased pathology	(103)

IL-1R1^−/−^	DSS	Increased pathology	(104)

	*C. rod*	Increased pathology	(104)

	T cell transfer colitis	Decreased pathology upon transfer of IL-1RI^−/−^ T cells into lymphopenic hosts Decreased Th17 cell survival	(101)

**IL-18R signaling pathway**			

IL-18^−/−^	–	Increased intestinal Th1 and Th17 effector cells Non-hematopoietic expression [intestinal epithelial cells (IEC)]	(49)

	DSS	Increased pathology	(33)

	*C. rod*	No effect on bacterial colonization	(104)
		Increased bacterial colonization Increased pathology	(103)

	*S*. Tm	No effect on intraepithelial load	(14)

	Rotavirus	Comparable viral load	(37)

IL-18Tg	DSS	Increased pathology	(123)

IL-18^ΔIEC^	DSS	Decreased pathology	(110)

IL-18^Δ/HE^	DSS	Decreased pathology	(110)

IL-18R^−/−^	–	Increased intestinal Th1 and Th17 effector cells Decreased intestinal Treg function	(49)

	*C. rod*	Increased bacterial colonization Increased pathology	(43)

IL-18r^ΔIEC^	DSS	Decreased pathology	(110)

IL-18r ^Δ/HE^	DSS	No difference in pathology	(110)

IL-18bp^−/−^	DSS	Increased pathology Increased goblet cell loss	(110)

IL-18bp^−/−^IL-18r^Δ/HE^	DSS	No difference in pathology	(110)

*C. rod, Citrobacter rodentisum; S. Tm, Salmonella Typhimurium; Treg, T regulatory cells*.

*Mutant strain: IL-18Tg, IL-18 transgenic: overexpression of IL-18; IL-18^ΔIEC^, IL-18-deficient IEC; IL-18^Δ/HE^, IL-18-deficient hematopoietic cells; IL-18r^ΔIEC^, IL-18R-deficient IEC; IL-18r ^Δ/HE^, IL-18R-deficient hematopoietic cells; IL-18bp^−/−^, IL-18 binding protein-deficient; IL-18bp^−/−^IL-18r^Δ/HE^, IL-18bp^−/−^ with IL-18R-deficient hematopoietic cells*.

Intestinal epithelial cells face a unique challenge as they constitute the first cellular border between the complex contents of the gut lumen and the largely sterile subepithelial compartment. This intestinal epithelial surface area is greatly increased by gland like invaginations called crypts, as well as projections of small finger like protrusions in the small intestine, known as villi. IEC are composed of various specialized cell types; enteroabsorptive cells, goblet cells, Paneth cells, neuroendrocrine cells, tuft cells, and stem cells. Due to the constant epithelial turnover, stem cells are responsible for replenishing any lost cells *via* Notch-mediated epithelial cell differentiation (72). Goblet cells secrete heavily glycosylated mucins which form a mucus matrix (73) into which Paneth cells secrete antimicrobial peptides (AMP) (74–76), together providing a physical and chemical barrier between the epithelial cell layer and the luminal contents. This barrier is further fortified by the secretion of IgA dimers into the mucus layer which act to sterically hinder any potential threats (77). In addition, goblet cells have been reported to deliver luminal antigens to subepithelial antigen-presenting cells enabling screening of the luminal contents (78). Thus, there are numerous antimicrobial mechanisms employed by the epithelium to limit access of potentially inflammatory stimuli.

During homeostasis, interactions with the microbial and dietary antigens induce a non-inflammatory IEC state that promotes immune tolerance. However, luminal content occasionally carries pathogenic microorganisms or toxic particles capable of causing mucosal damage and, in severe cases, systemic disease. Accumulating evidence suggests that the inflammasome plays a key role in modulating epithelial responses at the host–lumen interface. Data generated on purified IEC, *in situ* detection, or cell-specific ablation have revealed an expression of an array of inflammasome components within IEC including; NAIP, NLRC4, NLRP1, NLRP6, AIM2, caspase-1, caspase-4/5 (human), caspase-11 (mouse), Asc, and IL-18 (Tables 2 and 3) (79, 80). This review will discuss the functional importance of the inflammasome and its components within the context of epithelial cells and intestinal inflammation.

## Inflammasomes and Their Soluble Mediators in Intestinal Homeostasis

Inflammasome formation and caspase-1 activation lead to cleavage and secretion of the active forms of IL-1 family member cytokines, such as IL-1β and IL-18. These cytokines play a central role in immunity due to their diverse array of biological functions and broad range of target cells. IL-1β is a potent pro-inflammatory cytokine exerting a plethora of systemic and local effects. IL-1β promotes the recruitment of immune cells to the site of inflammation *via* induction of adhesion molecules and chemoattractants (81, 82). Stimulation with IL-1β promotes the activation and effector functions of dendritic cells, macrophages, and neutrophils (83). In addition, IL-1β plays a role in adaptive immunity driving T cell activation and survival (84), and acting in concert with other cytokines to promote Th17 cell differentiation (85). Due to these highly pro-inflammatory properties, IL-1β release is tightly regulated *via* a two-step process, namely, TLR-induced production of an inactive ~31–34 kDa precursor pro-IL-1β, followed by caspase-1 dependent cleavage and secretion of the active form (86).

Several clinical studies reported high levels of IL-1β production by the lamina propria mononuclear cells from active colonic lesions of IBD patients (87–89). IL-1β levels in the colon correlated with disease activity suggesting an important role for this cytokine in driving local inflammation (90, 91). Elevated colonic IL-1β levels are also characteristic of many animal IBD models (92–94), and strategies blocking IL-1β signaling were beneficial in ameliorating acute models of intestinal inflammation (95–98). Moreover, genetic alterations of key innate immune molecules, such as NOD2 and Atg16l1, resulted in over production of IL-1β by macrophages and enhanced susceptibility to dextran sodium sulfate (DSS)-mediated intestinal injury (99, 100). In addition, IL-1β augmented the recruitment of granulocytes and the activation of innate lymphoid cells during *Helicobacter hepaticus*-triggered intestinal inflammation, and IL-1R signaling in T cells controlled the early accumulation and survival of pathogenic Th17 cells in the colon during T cell transfer colitis (101). The role of IL-1β in promoting intestinal inflammation has also been confirmed in infection studies, as blocking IL-1β ameliorated pathology in both *Clostridium difficile*-associated colitis and *Salmonella* Typhimurium-induced enteritis (68, 102). However, alternative studies suggest a protective role for IL-1β during *Citrobacter rodentium* induced intestinal inflammation, as IL-1R1^−/−^ and IL-1β^−/−^ animals suffered from increased bacterial loads and pathology (Table 3) (103, 104).

Although IL-1β signaling appears to play a predominant role in mediating intestinal inflammation, IEC do not produce significant levels of IL-1β themselves (44, 105). Interestingly, stratified epithelia at other sites produce considerable amounts of IL-1β upon activation of their NLRP3 inflammasome (106, 107). The significance of differential IL-1β expression between epithelial cell types in distinct tissues remains incompletely explored. In the gut, it appears that lamina propria phagocytes constitute the main source of IL-1β during intestinal inflammation (101, 108).

In contrast, there is substantial evidence for the expression and secretion of IL-18 by the intestinal epithelium. Notably, at steady state in the intestine IEC appear to be the primary source of IL-18 (44, 49, 50, 109). The inactive 24 kDa precursor pro-IL-18 is constitutively expressed by IEC, primed for release upon inflammasome activation (44, 49, 50, 109, 110). Akin to IL-1β, IL-18 has been shown to induce a diverse array of immune responses. Originally termed IFNγ-inducing factor, IL-18 is typically considered a Th1 promoting cytokine due to its ability to elicit IFNγ production by T cells (111). However, in the presence of the correct co-stimuli, IL-18 can also drive Th2 cytokine production (112), or IL-17 production by γδ T cells (113). In addition, IEC derived IL-18 can drive perforin production by NK cells during enteric infection with *S*. Typhimurium, revealing an important role for IEC in coordinating acute mucosal responses (114).

Genome-wide association studies have linked mutations within the IL-18R1-IL-18RAP locus with susceptibility to IBD (115–117). Furthermore, increased IL-18 levels were detected in the biopsies of CD patients (50, 118). Using immunohistochemical analysis, IL-18 localized to the epithelium of non-inflamed regions, whereas in involved regions IL-18 was detected in cells morphologically described as macrophages (50). However, this altered IL-18 distribution was specific to CD, as UC patients displayed an epithelial distribution of IL-18 regardless of severity (50). Moreover, the bioactivity of mature IL-18 is regulated by the production of IL-18 binding protein, levels of which are also elevated in CD patients (119, 120). Thus, although the contributions of IL-18 to clinical intestinal inflammation remain unclear, evidence suggests that dysregulated IL-18 signaling could influence intestinal inflammation.

In murine models, different studies have drawn conflicting conclusions on whether IL-18 plays a predominantly pathogenic or protective role in intestinal inflammation. Early studies using biochemical inhibition of IL-18 signaling revealed a detrimental role for the cytokine in intestinal inflammation mediated by DSS (121, 122). Furthermore, overexpression of *IL-18* in IL-18 transgenic mice resulted in increased severity of DSS-mediated intestinal injury (Table 3) (123). Hyperproduction of IL-18 in mice deficient in Atg16l1, a key autophagy adaptor molecule, was also associated with increased susceptibility to DSS, a phenotype which was rescued by antibody-mediated blockade of IL-18 (100). This exacerbated inflammation associated with IL-18 may be due to its ability to induce pro-inflammatory effector T cell activation, even in the absence of T cell receptor engagement (111, 113, 124, 125). In fact, intestinal T cells express significantly greater amounts of IL-18R than those found in systemic lymphoid tissues, suggesting that they may be particularly sensitive to IL-18 signaling (49). Indeed, blocking IL-18 signaling protected mice against colitis mediated by transfer of naive T cells into lymphopenic hosts (126).

Conversely, independent studies using IL-18- and IL-18R-deficient mice revealed a beneficial role for IL-18 signaling during DSS colitis (Table 2) (127, 128). In addition, caspase-1^−/−^ animals were more susceptible to DSS-mediated colitis, which was associated with decreased epithelial cell proliferation and IL-18 secretion (51). This was corroborated by Zaki et al., who also observed increased susceptibility to DSS colitis in the absence of caspase-1 (Table 2) (52). Interestingly, this exacerbated phenotype could be rescued through administration of recombinant IL-18 (rIL-18), but not by adoptive transfer of myeloid cells, suggesting that IL-18 expression in the non-hematopoietic compartment was essential for protection (51, 52). Similarly, non-hematopoietic NLRP6 expression was found to be necessary to protect against DSS colitis, an effect that was again associated with impaired IL-18 production (33). In addition, deficiencies in NLRP6 were associated with a dominant dysbiosis (33) and decreased microbiota diversity (32), with rIL-18 treatment ameliorating this effect by increasing AMP production by IEC (32). Furthermore, a metabolomics screen identified potential microbiome-derived metabolites capable of modulating NLRP6 inflammasome activation and subsequent IL-18 secretion (32). Thus, deficiencies in NLRP6 expression are associated with reduced IL-18 production and the emergence of a dysbiotic microbiome that sensitizes mice to exacerbated DSS-mediated intestinal inflammation. In addition, deficiency in the cytosolic dsDNA sensor AIM2 also led to increased pathology upon DSS administration, which was again associated with decreased IL-18 signaling (Table 2) (42, 129).

In fact, DSS colitis is ameliorated in antibiotic treated genetically susceptible mice (33, 42, 98, 129–131), or exacerbated in mice receiving transfers of pathobionts (98), signifying the importance of the microbiota composition in this model. Microbiota sensing may also mediate protective effects against DSS colitis as evidenced by reports of exacerbated disease in germ-free mice (132) and Myd88^−/−^ mice (133). A key caveat of many studies using DSS colitis models in mice with genetic deficiencies is that they did not employ appropriate co-housing strategies to minimize any potential effects of the microbiota. As such, variations or “dysbiosis” in the microbiota may have occurred as a result of long-term microbial divergence due to extended isolation of breeding cohorts, as was reported for TLR-deficient mice (134). Therefore, studies in which inflammasome-deficient strains were compared to independent breeding cohorts of wild type mice must be interpreted with caution. In addition, these conflicting results emphasize the importance of using littermate controls to evaluate potential differences in susceptibility to experimental colitis in genetically modified mice.

Epithelium-derived IL-18 has also been implicated in protecting against infection-associated intestinal inflammation. For example, IL-18-deficient or IL-18R-deficient mice were more susceptible to colonization and inflammation upon infection with *C. rodentium* (Table 3) (43, 103, 109). Similarly, caspase1^−/−^ animals suffered from increased susceptibility to *C. rodentium* infection which was associated with increased inflammatory responses and decreased IL-18 secretion, suggesting a protective role for IL-18 in this model (103). Consistent with these findings, mice deficient in NLRP3 or Asc also suffered from exacerbated *C. rodentium* infection and pathology (43). Furthermore, non-hematopoietic cells were the source of this protective NLRP3 and Asc circuit, with strong Asc expression evident in the IEC (43). However, although *C. rodentium*-infected Asc^−/−^ animals almost completely lacked IL-18 in the intestine, the absence of NLRP3 did not affect IL-18 secretion (43). Thus, NLRP3 signaling may be mediating alternative protective pathways aside from IL-18 production (43). NLRC4 expression in IEC is also important for protection against *C. rodentium* induced intestinal inflammation, and NLRC4 deficiency was associated with decreased basal IL-18 levels and increased early pathogen colonization of the epithelium (25). Thus, the discrepancies in intestinal IL-18 production between the NLRP3- and Asc-deficient mice may be explained in part by compensation of the NLRC4 inflammasome in the absence of NLRP3 expression. Finally, NLRP6 inflammasome expression was also reported to protect against *C. rodentium* induced inflammation, and this was linked to effective mucin granule exocytosis by goblet cells (Table 2) (34). In addition, NLRP6 inflammasome formation and subsequent IL-18 secretion also enhanced AMP production by IEC (32). The non-redundant requirement for several NLR in protection from attaching and effacing pathogens like *C. rodentium* suggests that distinct NLR may mediate slightly different protective responses in IEC and/or that activation of NLR in additional cell types may contribute to epithelial protection. In addition, whether and how different inflammasomes interact during *C. rodentium* infection remains to be fully elucidated, although there is some evidence for the interaction of NLRP3 and NLRC4 inflammasomes during *S*. Typhimurium infection (135).

The epithelial protective effects of IL-18 may be explained by its roles in wound healing (127, 136) and in driving IL-22 (109), a cytokine important for AMP production and mucosal barrier integrity (137, 138). Of note, IL-22 expression has been shown to protect mice against several models of IBD (139, 140). In fact, administration of rIL-18 to IEC decreased their production of IL-22 binding protein allowing for greater amounts of IL-22 signaling (42). Interestingly, co-administration of IL-22 and IL-18 induced reprogramming of IEC gene expression, not observed with either cytokine alone, which correlated with protection against rotavirus infection, suggesting that these cytokines may act in concert in the intestine to promote antimicrobial responses (141). In addition, IL-18 has also been demonstrated to promote optimal T regulatory cells responses in the gut, with the lack of IL-18 associated with increased proinflammatory T effector cells (49).

Such studies have led to the conclusion that epithelial-derived IL-18 promotes barrier integrity and maintains a healthy microbiota, which contributes to protection against intestinal injury and inflammation. However, this function of IL-18 has been inferred from complete deletion of inflammasome components, as well as the cytokine itself, alongside bone marrow chimera studies. Recently, studies have been conducted using IEC-specific IL-18 knockouts (IL-18^ΔIEC^) (110) and IEC-specific caspase-1 knockouts (Casp1^ΔIEC^) (Tables 2 and 3) (142). These studies reported that caspase-1 activation and consequent IL-18 secretion by IEC during DSS colitis was associated with exacerbated inflammation and decreased goblet cell maturation (110, 142). These findings are somewhat surprising, as NLRP6 deficiencies were previously associated with both decreased IL-18 levels (33) and goblet cell mucus secretion (34), which led to increased susceptibility to DSS-mediated intestinal injury. In addition, several studies demonstrated that rIL-18 administration rescued inflammasome-deficient phenotypes from hypersusceptibility to DSS colitis (32, 47, 51, 52, 131). Considering these publications, the authors argue that extrapolation of IL-18 functions from mice fully deficient in inflammasome components should be interpreted with caution, as such deletions may affect the myeloid compartment beyond the scope of IL-18 production (i.e., there could be confounding effects on IL-1β production and pyroptosis). However, numerous bone marrow chimera experiments pointed to the importance of non-hematopoietic inflammasome expression in mediating protection against intestinal inflammation (25, 43, 51, 52, 129). As noted above, it is very likely that the microbiota is a key confounding factor, therefore repeating DSS colitis in IL-18^ΔIEC^ mice housed in alternative vivariums could help clarify the contribution of genotype versus microbiota. Clearly, further studies using mice with cell-type specific ablation of inflammasome components (or effector molecules) need to be carried out to better understand their diverse functions in IEC.

In addition to IL-1 family cytokines, inflammasome activation affects the release of alternative bioactive factors by immune cells. The alarmin high-mobility group box 1 (HMGB1) was originally identified as a nuclear DNA-binding protein. Upon infection or injury, inflammasomes were shown to mediate extracellular release of HMGB1 from stimulated immune cells triggering inflammation (143, 144). In the context of epithelial cells, LPS transfection of IEC led to HMGB1 release (46) and infection of gingival epithelial cells with *Fusobacterium nucleatum* drove release of HMGB1 alongside Asc and IL-1β secretion (107), suggesting that inflammasomes may be involved in the active secretion of HMGB1 from IEC. Caspase-1 activation has also been hypothesized to play a role in unconventional protein secretion of leaderless peptides such as IL-1α and FGF_2_ from macrophages (145). Others have postulated that AMP may be regulated *via* post translation modification by an effector downstream of inflammasome activation (146). The lipid inflammatory mediators, eicosanoids, have also been linked to inflammasome-dependent unconventional secretion (147, 148). In fact, the eicosanoid prostaglandin PGE_2_ was secreted by murine IEC upon NLRC4 inflammasome activation (24). Examination of the downstream soluble mediators of inflammasome activation, aside from IL-1β and IL-18, remains comparatively understudied in IEC compared to classical immune cells. Future work will need to address this by systematically examining the inflammasome-dependent secretome of activated IEC, and its downstream activities.

## Inflammasomes and Cell Death: Pyroptosis and Apoptosis

Inflammasome functional studies to date have largely focused on the secretion of downstream soluble mediators. However, there is much emerging interest in the role of inflammasome-dependent cell death, termed pyroptosis, an inflammatory form of cell death (149, 150). Pyroptosis takes place following engagement of “canonical” (caspase-1) or “non-canonical” (caspase-11) inflammasomes. “Non-canonical” triggering of pyroptosis occurs by intracellular LPS engagement with caspase-11 and has mainly been described in macrophages (151, 152). Identification of the “non-canonical” pathway followed from the finding that 129SvEv mice carried a passenger mutation that truncated the *caspase-11* gene (59), meaning that the original *caspase-1* knockout mice, which were generated on a 129SvEv background, were deficient in both *caspase-1* and *caspase-11*. Using caspase-11 complementation, Kayagaki et al. showed that macrophages underwent caspase-1-independent “non-canonical” cell death in response to several inflammasome activating stimuli, including Gram-negative bacteria such as *Escherichia coli, Vibrio cholerae*, and *C. rodentium*, as well as LPS co-treatment with cholera toxin subunit B (59). Subsequently, it was found that macrophages that were loaded with intracellular LPS activated caspase-11 and died by pyroptosis, and that mice lacking caspase-11 were protected from LPS-induced endotoxemia and pyroptosis (59–61). Finally, two independent studies identified caspase-11 as the key intracellular receptor for LPS (46, 153).

Caspase-11-driven pyroptosis has been shown to be key for protection against intracellular pathogens, particularly those that can escape from phagocytic vacuoles, such as *S*. Tm (151, 154, 155). However, studies with phagocytes and embryonic fibroblasts reported that caspase-1 “canonical” inflammasomes were required for efficient processing of IL-1β and IL-18, even in the context of direct caspase-11 activation, which was only able to lead to cytokine cleavage *via* indirect activation of caspase-1 (156–159). Nevertheless, caspase-11-dependent activation of IL-18 has also been reported, for instance, cecal tissue explants from *S*. Tm-infected *caspase-11*-deficient mice were also defective in IL-18 but not IL-1β secretion (44). Furthermore, colonic tissue explants from *C. rodentium*-infected *caspase-11*-deficient mice also had decreased IL-18 secretion (160). This caspase-11-dependent IL-18 processing was proposed to occur in IEC, contrary to the caspase-1-dependent cleavage of IL-18 and IL-1β observed in myeloid cells (161).

The importance of “canonical” and “non-canonical” inflammasomes may vary depending on the nature and characteristics of the pathogenic threat and the cell types involved. For example, upon challenge with flagellin-deficient *Salmonella*, caspase-1-deficient macrophages died in a similar manner to WT macrophages, whereas caspase-11-deficient macrophages were resistant to cell death (158, 161). In contrast, both caspase-1 and caspase-11 were required for cell death in macrophages infected with WT *Salmonella* (158). This highlights the fact that *Salmonella* can activate both the “canonical” inflammasome, through flagellin–NAIP–NLRC4 interactions, and the “non-canonical” inflammasome, through direct LPS–caspase-11 interactions (Figure 1). The complementary roles of “canonical” and “non-canonical” inflammasomes are especially important in the context of bacterial infections. Bacterial evasion strategies can counteract inflammasome responses, such as inhibition of epithelial caspase-11 *via* NleF, a type 3 secretion system effector protein produced by *E. coli* and *C. rodentium* (160). In a caspase-11-deficient scenario, however, pyroptosis may still proceed due to intact caspase-1 activation, highlighting potential redundancy of these two caspases (162). It seems logical that the intestinal epithelium, as a first line of defense, would have intrinsic mechanisms to warn the immune system of an invading threat. Indeed, as noted above, caspase-11 in mice (an ortholog of human caspases-4/5) is important for the recognition and clearance of *S*. Tm, and mice lacking caspase-11 harbor increased loads of *S*. Tm in the intestinal epithelium (14, 44). Furthermore, siRNA knockdown of caspase-4 in human colonic IEC led to reduced cell death upon *E. coli, S*. Tm, and *Shigella flexneri* infection (44, 163), and this was accompanied by increased *S*. Tm intracellular load, and reduced IEC shedding (44, 161).

Recent studies, in addition to highlighting the importance of “non-canonical” inflammasomes in innate immune defense in IEC, have also shed some light on the mechanisms involved in IEC-intrinsic restriction of *S*. Tm invasion. The innate immune sensor NLRC4 and its NAIP adaptors were shown to be essential for the extrusion of infected IEC into the intestinal lumen following *S*. Tm challenge of streptomycin-treated mice (14). IEC extrusion may represent a cell-intrinsic defense mechanism that serves to limit the rate of pathogen colonization of the intestinal epithelium. In this study, it was unclear whether IEC extrusion was linked to pyroptosis, as plasma membrane integrity of extruded enterocytes seemed to be maintained (14). However, by using an inducible construct to drive the expression of NLRC4 specifically in the intestinal epithelium, Rauch et al. showed that IEC-specific NLRC4 activation by FlaTox (*Legionella pneumophila* flagellin fused to the N-terminal domain of *Bacillus anthracis* lethal factor to drive cytosolic delivery) was sufficient to drive pathology, IEC death and IL-18 release (24).

Moreover, in agreement with the findings of Sellin et al., FlaTox activation of NLRC4 in IEC also limited *S*. Tm colonization of intestinal tissues and drove IEC death and extrusion (14). However, FlaTox-induced expulsion of IEC was accompanied by lytic cell death with plasma membrane permeabilization, resembling pyroptosis (24) (see Table 4 for morphological features of pyroptosis). From these studies, it becomes clear that, upon NLRC4 activation, IEC can undergo cell death and expulsion from the intestinal epithelium. In parallel, experiments in which caspase-1 expression was selectively induced in IEC, it was found that caspase-1 could drive pyroptosis in response to NLRC4 activation by FlaTox. On the contrary, caspase-1-deficient IEC did not undergo lytic cell death but were expelled from the epithelial layer with intact plasma membranes, indicating that caspase-1 was required for pyroptosis but not for IEC extrusion (24). Furthermore, they also observed that caspase-1-independent IEC extrusion following NLRC4 activation was dependent on both Asc and caspase-8 (24). Taken together, these studies show that various inflammasome-dependent responses are triggered in IEC during *S*. Tm infection, and these encompass activation of NLRC4, caspase-1, caspase-11 and possibly caspase-8 (14, 24, 44) (see Figure 1). However, it is unclear how the different inflammasome responses are regulated in IEC and if they are redundant, complementary, or interdependent. In addition, further studies are required to better define the precise kinetics and interconnections between downstream responses, such as IEC expulsion and cell death.

**Table 4:** Characteristic features of different cell death pathways.

Characteristic of the dying cell	Apoptosis	Necrosis	Pyroptosis	Necroptosis
DNA fragmentation	+ (171–173)	+/− (171, 172)	+ (174–177)	? (See necrosis)
Nuclear condensation	+ (171–173)	(172, 178)	+ (179)	− (172, 180)
Nuclear integrity maintained	− (171–173)	+ (171, 172)	+ (181)	+ (172, 180)
Cell swelling	− (171–173)	+ (171, 172)	+ (175)	+ (172, 180)
Lysis/membrane permeability	− (171–173)	+ (171, 172)	+ (175)	+ (178)
Membrane blebbing and shedding	+ (171–173)	− (171, 172)	− (182)	? (See necrosis)
Membrane pore formation	−	−	+ (183–185)	+ (186, 187)
DAMP release	−	+ (188)	+ (179)	+ (178)
IL-1β and IL-18 release	−	−	+ (179)	−
Main caspases	casp-3 and casp-7	Non-caspase mediated	casp-1 and casp-11 (mouse) casp4 and casp-5 (humans)	Non-caspase mediated (189)

*+, present; −, absent; +/−, present to a limited degree; ?, not yet assessed*.

*GsdmD, gasdermin D; DAMP, danger-associated molecular pattern*.

The detailed role of pyroptosis *in vivo* remains largely unexplored due to limited knowledge of the downstream targets of caspase-1 and caspase-11 culminating in cell death. Recently, however, gasdermin D (GsdmD) was identified as a direct downstream target of caspase-1 and caspase-11 and was shown to be required for pyroptosis upon “canonical” and “non-canonical” inflammasome engagement (150, 183, 184, 190, 191). Indeed, upon GsdmD cleavage by caspase-1 or caspase-11, its ~30 kDa N-terminus embeds itself in the plasma membrane, forming 10–14 nm pores and ultimately leading to lytic cell death (184, 185, 192). Interestingly, GsdmD is highly expressed in the intestinal epithelium, suggesting that GsdmD may also be involved in pyroptosis in IEC (192, 193). Consistent with this hypothesis, IEC pyroptosis in response to *in vivo* administration FlaTox did not proceed in gasdermin D-deficient mice (24). A very recent study using a mouse model of rotavirus infection reported that activation of a novel NLR inflammasome that recognizes viral dsRNA, NLRP9b, contributed to the restriction of rotavirus replication in IEC organoids, at least partly through gasdermin D-induced pyroptosis (37). Furthermore, mice deficient in either GsdmD or NLRP9b displayed increased susceptibility to rotavirus infection *in vivo* (37). Collectively, these reports suggest that different IEC inflammasomes converge on GsdmD-induced pyroptosis to restrict pathogen load in infected IEC.

Pyroptosis shares a number of morphological features with both apoptotic and necrotic forms of cell death (Table 4). Akin to necrosis, in pyroptosis, nuclear integrity is maintained, and the cell undergoes cytoplasmic swelling due to membrane permeabilization that ultimately terminates in cell lysis (174, 179). Akin to apoptosis, pyroptotic cells exhibit DNA fragmentation and are TUNEL positive, as well as presenting nuclear condensation (174–176, 179). Before the acknowledgment of pyroptosis as a new form of cell death (57), its similarities to necrosis and apoptosis led researchers to attribute inflammasome-driven cell death to only apoptosis and/or necrosis (174, 176, 179). It is partly for this reason that the interconnections between the different types of cell death upon inflammasome activating stimuli remain poorly understood. The discovery of GsdmD as a key player in pyroptosis should help elucidate the molecular pathways involved (150, 191, 192).

In addition to pyroptosis, inflammasome responses in various cell types have also been linked to apoptotic cell death. For instance, ectopic expression of NLRC4 and Asc in HEK293T cells (which lack caspase-1) showed that these molecules can engage with caspase-8 to drive apoptosis (194). Furthermore, both apoptosis and pyroptosis have been observed in macrophages following NLRP3 or AIM2 activation (194, 195). Interestingly, macrophages lacking GsdmD were reported to undergo cell death upon LPS plus *S*. Tm or nigericin treatment, through a poorly defined mechanism that was independent of caspase-1, were delayed compared to pyroptosis, and had some features of apoptosis (192).

The literature also suggests some cross-regulation between pyroptosis and apoptosis as THP-1 cells treated with etoposide, an apoptosis inducing drug, resulted in the cleavage of GsdmD into a ~43 kDa fragment, different from the 30 kDa fragment observed in pyroptosis, that occurred independently of caspase-1 (196). The generation of the 43 kDa fragment was observed upon caspase-3 and -7 activation during apoptosis. This suggests that the apoptosis and pyroptosis pathways may compete for the same substrate and that cells may not be able to simultaneously undergo both forms of cell death. The authors speculated that the alternative cleavage of GsdmD by apoptotic caspases-3 and -7 may prevent apoptotic cells from becoming pyroptotic, thus maintaining and immunologically silent cell death (196).

Other studies suggest that differing thresholds may operate between the two cell death pathways following inflammasome activation. For example, in macrophages, for caspase-8 dependent apoptosis to occur upon AIM2 activation, the concentrations of DNA required were much lower than for pyroptosis (195). Under low stress levels, it would be desirable to deal with the invading threat in an immunologically silent way to avoid hyper inflammation, thus apoptosis would be favored. However, when the threat is high, an inflammatory response could help deal with the microbial insult, therefore pyroptosis may be beneficial. However, it is important to stress that it remains to be demonstrated if this threshold-dependent decision controls differential cell death pathways following inflammasome activation *in vivo* and in cells other than macrophages.

## Linking Inflammasome Effector Mechanisms

Both IL-1β and IL-18 lack signal peptides and therefore are not secreted through the conventional ER–Golgi pathway (197–199). For IL-1β, the better described cytokine of the two, several routes of release have been proposed, including secretory lysosomes, exosomes, and microvesicles (200–204). The secretory exosome pathway was proposed through the observation that IL-1β in monocytes was localized in endosomal-like vesicles that are normally targeted for degradation, but can be redirected to the extracellular space (202, 205). In addition, microvesicle-mediated rapid secretion was proposed after observing vesicles associated with bioactive IL-1β as early as 2 min post-ATP stimulation in activated monocytes (203). However, studies on these secretory routes were often contradictory and employed different cell systems, thus these models of secretion remain controversial (206). The mechanisms of secretion of IL-18 are generally assumed to follow the mechanisms of IL-1β secretion but are much less investigated.

However, pyroptosis has now been proposed to be responsible for the release of IL-1β and IL-18 to alert the immune cells of the imminent danger, leading to the onset of inflammatory responses (207). This was first suggested by the observation of caspase-1-dependent pores in the plasma membrane of *Salmonella*-infected macrophages, ultimately leading to cell swelling and osmotic lysis (175). This was supported by more recent studies of ATP-stimulated BMDM, in which pharmacological inhibition of membrane permeabilization—a hallmark of pyroptosis—abolished IL-1β secretion, but not processing (200). The recent discovery of GsdmD and its requirement for pyroptosis offers a potential mechanistic explanation linking pyroptosis and cytokine secretion. Both caspase-1 and -11 are able to cleave GsdmD, releasing the active N-terminus that mediates pore formation and lytic cell death (184, 185, 191, 192) (Table 3). Consistent with the concept that pyroptosis facilitates cytokine secretion, macrophages lacking GsdmD exhibit defective IL-1β secretion in response to various “canonical” and “non-canonical” inflammasome activators, including intracellular LPS, Gram-negative bacteria and nigericin (150, 185, 191, 192). However, there is also evidence in the literature of IL-1β release in the absence of cell death in peritoneal macrophages, human monocytes, and neutrophils (208, 209). In particular, neutrophils were able to secrete IL-1β in response to *Salmonella* infection through a mechanism that was dependent on NLRC4 and caspase-1 but was independent of cell lysis (210). The mechanisms of secretion of inflammasome-processed cytokines may therefore be dependent on the cell type and the nature of the activatory signals.

It is again important to emphasize that inflammasome effector responses have largely been studied in leukocytes, particularly phagocytic cells. Whether the discoveries made in these cell types are applicable to tissue cells, including IEC, remains to be determined. For instance, classical activation of the inflammasome has long been viewed as a two-step process, starting with the transcriptional regulation of the inflammasome components. Thus, caspase-11 induced cell death in macrophages was dependent on priming by TLR4 ligands through TRIF, but not on Myd88 signals (157, 158, 211). Indeed, LPS administration in mice, rapidly induced *caspase-11* expression in various tissues including thymus, spleen, and lung (161, 212). Conversely, IL-1β release in phagocytic cells depended on Myd88-mediated transcriptional priming (3, 213). These requirements appear to be somewhat different in the intestinal epithelium, for example, although TLR4 signaling is downregulated in IEC (214), caspase-11-dependent responses still occur. This suggests that *caspase-11* is constitutively expressed in the intestinal epithelium and can be rapidly activated upon pathogen invasion (48). This “ready-to-go” phenotype of IEC inflammasome components is further supported by the observations that *NLRC4* and *pro-IL-18* are constitutively expressed by IEC and may not require priming (25, 49). Furthermore, the constitutive colonization of commensal Gram-negative bacteria in the intestine could explain the constitutive elevated expression of *caspase-11* and *IL-18* in the gut compared to other tissues (www.proteinatlas.org) (161).

It is also worth noting that during homeostatic conditions, and thus in the absence of inflammation, the inflammasome-dependent cytokine IL-18 is released from IEC and is believed to have functions in epithelial repair, proliferation and maturation (33, 34). The mechanisms of secretion of IL-18 by IEC during homeostatic conditions are not well understood and whether pyroptosis occurs in IEC under physiological conditions *in vivo* remains to be determined (215). Although there is increasing evidence that IEC-intrinsic inflammasome activation plays a key role in early innate defense against pathogens that target the intestinal epithelium (14, 24, 25, 43, 44), much remains to be learned on how inflammasomes and their downstream effector responses are regulated in IEC. Due to their constitutive exposure to microbial PAMP, inflammasome circuits and thresholds in IEC may be quite different to those primarily identified in macrophages and dendritic cells. Nevertheless, the constitutive secretion of IL-18 by IEC indicates that inflammasomes are active under homeostatic conditions in the intestinal epithelium. However, the precise signals or thresholds that determine when this may be superseded by the induction of pyroptosis or alternative cell death pathways remain to be determined. For example, it will be important to assess the role of GsdmD in IL-18 secretion and IEC turnover during steady-state conditions. Furthermore, it will also be vital to understand how inflammasome responses in IEC are modulated during pathogenic attack or during inflammatory conditions, where an optimal balance between apoptosis, pyroptosis, and cytokine release may be required to control potential pathogens and restore homeostasis.

## Conclusion and Perspectives

High expression levels of many inflammasome proteins are enriched in the steady-state intestinal mucosa implicating their importance in barrier maintenance and immune monitoring. The spatial location of the IEC, directly facing the lumen, in combination with their primed phenotype, implies that inflammasomes are key sensors of intestinal insults. Indeed, as discussed throughout this review, deletion of these components is primarily associated with increased susceptibility to injury and infection. Thus, we can conclude that epithelial inflammasomes are critical for a healthy gut, both at steady state and during acute infection or injury. However, the molecular mechanisms orchestrating epithelial inflammasome activation remain incompletely understood, representing a key area for further research.

Frustratingly, the literature contains numerous examples of conflicting data pertaining to the functional impact and cellular sources of inflammasome components in various models of intestinal infection and inflammation. To better define these, the field needs to implement stringent lines of investigation that properly control for key environmental factors. Variation of the intestinal microbiome is likely responsible for most of the inconsistent findings reported the literature. For example, recent studies have identified protozoa (216) and microbial metabolites (32) as novel environmental factors capable of influencing inflammasome activation in the intestinal epithelium and in modulating susceptibility to intestinal inflammation. Therefore, standardized use of littermate controls for *in vivo* experiments should be implemented to circumvent misinterpretations resulting from differences in microbiota composition and baseline mucosal immune activation across distinct breeding cohorts. Furthermore, as different animal facilities will harbor their own distinct microbiotas, it would be advantageous if key experiments were reproduced in different vivariums.

To further assess the specific locations important for inflammasome function, tissue- and cell-specific deletion approaches represent an important approach, for example, the IL-18^ΔIEC^ line specifically lacking IL-18 production in IEC (110). In addition, complementary studies using inducible knockouts will be useful for understanding acute responses while ruling out any developmental disadvantages. The increasing application of primary intestinal epithelial “organoid” cultures will complement the *in vivo* genetic approaches, enabling analysis of acute responses, as well as offering a tool for molecular manipulation of IEC (217). Moreover, transitioning from murine studies into humans will be bolstered by these new *ex vivo* techniques (218).

Murine bone marrow-derived macrophages have served as the gold standard for a majority of inflammasome research, contributing significantly to our understanding of inflammasome signaling and effector responses. However, it is likely that IEC inflammasomes are regulated differently to classic hematopoietic cells, due to the unique intestinal environment. Thus, we need to address how inflammasome activation and regulation in IEC differs from that described in myeloid cells and the resulting implications. For example, we can already surmise from the literature that IEC produce comparatively little IL-1β (44, 105) and constitutively express IL-18 (49). It is likely that within IEC there is a different composition of inflammasome machinery to tailor their immune responses. In addition, IEC could be capable of producing other potential secretory factors besides IL-18 upon inflammasome activation, for example, prostaglandin production by IEC was recently associated with NLRC4 activation (24). The signaling circuitry and relationship between different effector responses also needs to be elucidated. For example, are there distinct activation thresholds or can different inflammasome components work in concert, as has been described for NLRC4 and NLRP3 during *S*. Typhimurium infection of macrophages (219).

Our understanding of what specific agonists activate IEC inflammasomes is limited and warrants further investigation. Aside from microbial signals, how do dietary antigens interact with the intestinal epithelium? Evidence already exists for the capacity of dietary ligands to induce inflammasome activation [e.g., high fat and high cholesterol diets (79)] or dampen inflammasome activation [e.g., ketones (220)]. However, further investigation is required to delineate whether these dietary factors act directly and/or indirectly (e.g., through modulation of the microbiota) (30). Indeed, a recent study reported that a high fiber diet conferred protective effects in the DSS colitis model both by reshaping the gut microbiota and by increasing release of SCFAs that activated NLRP3 inflammasomes in a non-hematopoietic cell population.

Finally, inflammasome activation in IEC has been described to result in IEC extrusion and cell death (14, 24). Further investigation needs to be carried out into the role of pyroptotic cell death in mucosal immune responses. The regulation of different forms of cell death in IEC and the consequences for infection or inflammatory diseases also requires further characterization. For example, does too little IEC death result increased potential for invasive infection due to lack of cell extrusion and does too much IEC death perpetuate unnecessary inflammation? Finally, what function does dysregulated inflammasome activation and pyroptosis play in IBD? IBD patients are known to have necrotic lesions and increased levels of IL-18 and IL-1β in the inflamed intestine, but their relative contributions to chronic intestinal pathology remain incompletely understood.

Despite these challenges and limitations understanding gut-associated inflammasome signaling, its role in regulating dietary–microbiome–host immune interactions constitutes a critical component in maintaining homeostasis and mediating various immune-mediated disorders. Encouragingly, the identification of small molecules capable of targeting specific inflammasome components (44) could represent an opportunity for novel clinical interventions to tackle these currently incurable disorders.

## Author Contributions

All authors listed have made a substantial, direct, and intellectual contribution to the work and approved it for publication.

## Conflict of Interest Statement

The authors declare that the research was conducted in the absence of any commercial or financial relationships that could be construed as a potential conflict of interest.
